# Ethylenediaminetetraacetate functionalized MgFe layered double hydroxide/biochar composites for highly efficient adsorptive removal of lead ions from aqueous solutions

**DOI:** 10.1371/journal.pone.0265024

**Published:** 2022-03-03

**Authors:** M. T. Amin, A. A. Alazba, M. Shafiq

**Affiliations:** 1 Alamoudi Water Research Chair, King Saud University, Riyadh, Kingdom of Saudi Arabia; 2 Department of Environmental Sciences, COMSATS University Islamabad, Abbottabad, Pakistan; 3 Agricultural Engineering Department, King Saud University, Riyadh, Kingdom of Saudi Arabia; Lappeenranta-Lahti University of Technology (LUT University), FINLAND

## Abstract

The application of layered double hydroxides (LDHs) of MgFe and its composites with biochar of *Eucalyptus camdulensis* (Eb) and ethylenediaminetetraacetic acid (EDTA) was explored in a batch study to mitigate toxic lead ions (Pb^2+^) from synthetic wastewater solutions. SEM images revealed that MgFe/LDH composites with Eb were successfully formed, while FTIR spectra confirmed the successful adsorption of Pb^2+^ onto the MgFe/LDH and composite adsorbents. Batch equilibrium was attained after 60 min, then the adsorption capacity gradually increased. An increase in adsorption capacity (and a 60% decrease in the percentage removal) was observed by increasing the initial Pb^2+^ concentration, and the highest value was 136 mg g^-1^ for MgFe/LDH-Eb_EDTA. A 50–60% increase in both the adsorption capacities and percent removal was seen in the pH range of 2–6. The second-order kinetic model had a nearly perfect fitting, suggesting that chemisorption was the mechanism controlling adsorption. The Langmuir isotherm model best presented the adsorption data, suggesting that the Pb^2+^ adsorption was monolayer, and predicted a better affinity between the adsorbent surface and absorbed Pb^2+^ for MgFe/LDH-Eb_EDTA in comparison to the other two adsorbents. The D–R isotherm suggested that the adsorption system was physical based on *E* values for all three adsorbents, while the Temkin isotherm model suggested that Pb^2+^ adsorption was heterogeneous. Finally, the Sips and R–P isotherms predicted that the adsorption of Pb^2+^ on the surface of the adsorbents was homogeneous and heterogeneous.

## 1. Introduction

The increase in heavy metal toxicity in natural freshwater reservoirs due to industrial growth has recently attracted much attention [1,2]. Lead (Pb), copper (Cu), arsenic (As), and cadmium (Cd) are very dangerous and highly toxic heavy metals that are typically found in industrial wastewater due to their tendency to bioaccumulate through the food chain; as a result, they cause harmful effects on livestock, aquatic life and human health [3–5]. Lead ions (Pb^2+^) are among the most common heavy metal ions with carcinogenic features and are often present in tannery, electroplating, metallurgical processes, pesticides, chemical processing, matching, photographic film, battery recycling processes, explosives, paint and pigment, and refinery wastewater [6]. Exposure to Pb^2+^ may result in severe poisoning in the liver and kidneys, as well as problems in blood pressure, mental retardation in children, reproductive system damage, and central nervous system damage [7,8]. Therefore, the unregulated disposal of effluents into fresh water supplies without treatment may cause shortages of fresh drinking water that is safe and clean. It has already been reported that approximately 25 million deaths worldwide are caused by the consumption of polluted drinking water [9]. Governments/policy-makers should, therefore, impose strict regulations on the management of industrial effluents to safeguard humanity from this serious danger. Therefore, researchers and scientists should come forward and help industries establish cheap, efficient, and eco-friendly methods for removing aqueous Pb^2+^ or other contaminants from industrial wastewater before being discharged into land and natural aquatic systems.

Many techniques, including chemical precipitation, ion exchange, electrocoagulation, reverse osmosis, solvent extraction and adsorption, have been established by different scientists for wastewater treatment [10]. Nevertheless, compared to conventional wastewater treatment technologies, adsorption is considered to be a very effective method due to its low cost, simplicity in implementation/operation, separation efficiency, and higher potential to adsorb heavy metals from low to higher concentrations [11–13]. Hence, choosing the most suitable and effective adsorbent with the highest stability, selectivity, and efficiency is very important. Various types of low-cost and efficient adsorbents, such as activated carbon, biochar, silica, zeolite, industrial byproducts, agricultural wastes, polymeric materials and clay minerals, and composite materials have been developed and studied conventionally to extract inorganic and organic contaminants from industrial wastewater [14–21]. However, this area of research has not yet been fully explored and needs further study. Therefore, there is an ongoing initiative to produce a high-quality and affordable adsorbent material that can be easily separated after being adsorbed from aqueous solution to prevent secondary contamination [22].

Layered double hydroxides (LDHs) have recently been found to be very efficient adsorbents because of their high surface area, low cost, large interlayer spaces (porosity), heat resistant structure, and large number of exchangeable anions with high anionic exchange capacities, making them excellent and desirable materials for use in adsorption [23,24]. The general formula of LDH is [M^2+^_1−*x*_M^3+^_*x*_(OH)_2_]A^*n*−^_*x*/*n*_·yH_2_O, where M^2+^ and M^3+^ are divalent and trivalent metal ions, respectively. A^n-^ represents an anion, and x is the ratio of M^3+^/(M^2+^ +M^3+^) [25]. The main property of LDH is that the anion present in the interlayer can be easily exchanged by the other anions so that LDHs can be used to extract a number of inorganic anions, such as phosphate, arsenate, bromate and fluoride, from aqueous solution [26–28]. Studies are being performed to increase the efficiency of LDH materials by intercalating LDH with chelating agents such as maltase, glutamate, and citrate, and an excellent heavy metal extraction from wastewater was achieved due to the formation of chelating complexes [29]. Some ongoing studies are also developing a combination of LDH with porous and functional materials such as agricultural or industrial porous carbon (biochar, activated carbon) that is waste-based, as well as graphene or graphite [30]. Hence, the present study aimed to obtain more active adsorption sites on the surface of LDH by mixing it with *Eucalyptus camdulensis* (*Ec*) tree bark waste, which can be used as a source of porous green carbon, and ethylenediaminetetraacetic acid (EDTA), which is a very popular and powerful chelating material with abundant functional groups (amino and carboxyl). It was hypothesized that the addition of *Ec* (Eb) biochar and EDTA with LDH can possibly generate a stable and highly efficient adsorbent that can effectively remove lead ions from wastewater.

The aim of this work was to develop and characterize LDHs of MgFe and its composites with Eb (MgFe/LDH-Eb) and with Eb and EDTA (MgFe/LDH-Eb_EDTA) and to study their potential capacity to remove toxic Pb^2+^ from synthetic wastewater solutions based on the stability of chelates formed between EDTA and Pb^2+^. The endeavor of comparing the performance of the simple and composite adsorbents, that has not been performed previously, is the focus of this work so that an efficient treatment system can be developed for aqueous systems. Additionally, the effects of pH, contact time, and initial concentration of Pb^2+^ and the amount of adsorbent on the adsorption of Pb^2+^ onto the synthesized adsorbents were examined. Isotherm and kinetic models were applied to the adsorption data to gain knowledge on the Pb^2+^ removal process.

## 2. Materials and methods

### 2.1. Chemical and adsorbent preparations

All the chemicals used in this study were of analytical reagent grade, including the lead nitrate that was used to prepare the stock solution (1000 mg L^-1^) of the model contaminant by dissolving 1.0 g of it (Pb(NO_3_)_2_, Tianjin Benchmark Chemical Reagent Co., Ltd. Tianjin, China) in 1.0 liter of deionized water. The prepared stock solution was further diluted to prepare working solutions with different initial concentrations of Pb^2+^ using deionized water. To maintain and adjust the solution pH as necessary for each batch test, 0.1 M NaOH and 0.1 M HNO_3_ were used.

Eb was prepared as reported previously [31], using *Ec* waste obtained from local areas around the city of Riyadh, Kingdom of Saudi Arabia. The collected waste was washed rigorously and thoroughly dried in an open atmosphere, and then the oven-dried (at 80°C for 6 hours) product was crushed in a ball mill to obtain an average particle size of nearly 5.0 mm. Finally, a box furnace (Nabertherm, B-150, Germany) was operated for 3 h at 600°C for the pyrolysis process to achieve biochar with an average particle size of 50–70 μm.

Magnesium nitrate hexahydrate (Mg(NO_3_)_2_·6H_2_O) and iron(III) nitrate nonhydrate (Fe(NO_3_)_3_·9H_2_O) were used to prepare the LDH of MgFe with a Fe^2+^/Mg^3+^ ratio of 1:2. A simple coprecipitation method was adopted in which both solutions were agitated in deionized water by maintaining the solution pH at 10.0, and ammonia was added dropwise with vigorous stirring to form the precipitate in approximately 30 min.

Composites from the prepared LDH of MgFe with Eb and Eb_EDTA were prepared by adding 3 and 5 g of Eb and EDTA, respectively, to 4 and 5 g (20% and 25%) of Mg(NO_3_)_2_·6H_2_O and Fe(NO_3_)_3_·9H_2_O solutions, respectively. The slurry was filtered and washed several times using double distilled water until the neutral pH became neutral. The resultant solutions were finally dried at 60°C for 24 h in an ordinary oven.

### 2.2. Methodology and batch testing

#### 2.2.1. Characterization

Scanning electron microscopy (SEM), energy dispersive X-ray (EDX) technique and Fourier transform infrared (FTIR) spectrometry were used to investigate the morphology and to obtain insight into the chemical composition of the MgFe/LDH and composite adsorbents before and after the heavy metal ions being studied were adsorbed. A quick comparison among the LDH, Eb and composite adsorbents (MgFe/LDH-Eb and MgFe/LDH-Eb_EDTA) was performed based on the pore structure and specific surface area measurement using Brunauer–Emmett–Teller (BET) analysis.

#### 2.2.2. Adsorption experiments

To obtain the necessary amount of each adsorbent in different batch tests, appropriate amounts of MgFe/LDH and its composites were measured based on the volume of the tested solution and initial Pb^2+^ concentrations. For the batch experiments, 50 or 100 mL of the suspensions were placed inside conical flasks and agitated at 220 rpm using a temperature-controlled (30°C) shaker (Wise Cube orbital, Wisd. ThermoStable IS-20; Daihan Scientific Co. Ltd., South Korea). After being agitated for a predetermined contact time, 5 mL of the sample was filtered through 0.45 μm (Whatman™) filters and inserted into a flame atomic absorption spectrometer (FAAS, Thermo Scientific, ICE 3000 Series, Cambridge, United Kingdom) to measure the residual metal concentration (*C*_*t*_, mg L^-1^). The adsorption capacity (*q*_*t*_, mg g^−1^) of the adsorbent and the percentage of Pb^2+^ removal was obtained using the initial (*C*_*0*_, mg L^-1^) and residual metal concentrations using Eqs (1) and (2), respectively. *V* (L) and *m* (g) represent the volume of the solution and the weight of the adsorbent, respectively.


$$Adsorption\;capacity=\left(\frac{C_{0}-C_{t}}{m}\right)\;V$$ (1)

$$Removal\;(\%)=\left(\frac{C_{0}-C_{t}}{C_{0}}\right)\times 100$$ (2)

Batch tests for optimization of parameters (contact time, solution pH, amount of adsorbent and initial Pb^2+^ concentration) were conducted by taking samples at least thrice while their average values are presented in Figs 3 and 4. The one-way analysis of variance was performed with the least significant difference of means at p = 0.01. Additional samples were taken whenever the difference between two measurements exceeded by 5%. The measurement of the equilibrium contact time was carried out at different initial concentrations (20–50 mg L^-1^) of Pb^2+^. For this purpose, the amount of the adsorbent was fixed at 0.3 g, while the solution pH was maintained at 6.0 over a contact time range of 1–300 min. The optimum initial Pb^2+^ concentration was determined by performing batch tests in the range of 20–100 mg L^−1^. The analysis was carried out at an optimized contact time of 60 min with a fixed amount of the adsorbent and solution pH of 0.3 g and 6.0, respectively. Batch tests for optimizing the solution pH were conducted in the range of 2–9, as shown in Fig 4B. For this purpose, 0.3 g of the adsorbent was added to the solution with an initial Pb^2+^ concentration of 40 mg L^−1^, and the samples were withdrawn after 60 min of contact time. Among the other important batch parameters, the amount of the adsorbent was also optimized due to its direct effects on the cost of the adsorption process. The adsorption performance was tested with the amount of adsorbent in ranges of 0.05–0.5 g by fixing the solution pH and initial Pb^2+^ concentration at 6.0 and 40 mg L^−1^, respectively, while suspensions were agitated for 60 min of contact time. Finally, the desorption experiments at different initial solution pH and temperatures were performed by using 0.1 M hydrochloric acid as an eluent in addition to a mild heat treatment inside an oven for 24h.

## 3. Results and discussions

### 3.1. Characteristics of the adsorbents

#### 3.1.1. Scanning electron microscopy analysis

**Fig 1:**
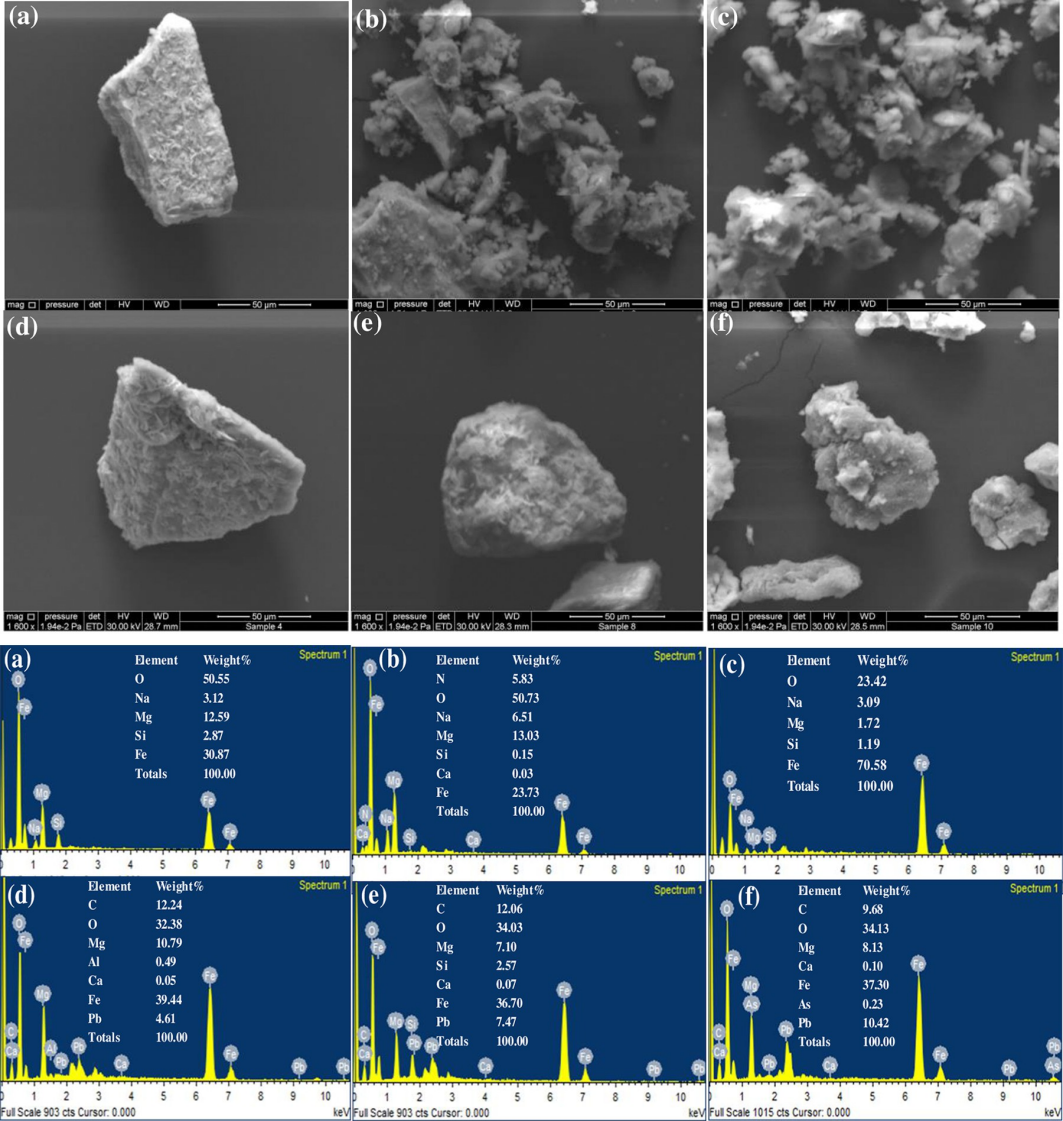
**a.** SEM images before and after adsorption of Pb^2+^; MgFe/LDH (a, d), MgFe/LDH-Eb (b, e) and MgFe/LDH-Eb_EDTA (c, f). **b.** EDX spectra before and after adsorption of Pb^2+^; MgFe/LDH (a, d), MgFe/LDH-Eb (b, e) and MgFe/LDH-Eb_EDTA (c, f).

The intercalation performance and surface structure of MgFe/LDH, its composite with the biochar of *Ec* (MgFe/LDH-Eb) and EDTA-functionalized composite adsorbent (MgFe/LDH-Eb_EDTA) before and after adsorption of Pb^2+^ ions were investigated by SEM observations, as depicted in Fig 1. The images of MgFe/LDH before and after Pb^2+^ adsorption are displayed in Fig 1A & 1D. Clumping of flowers, such as MgFe/LDH ultrafine nano glitter microspheres that were entangled with each other, can be clearly seen in the images [32,33]. Fig 1B & 1E shows SEM images of the MgFe/LDH-Eb composite before and after adsorption of Pb^2+^. The porous and rough surface of biochar particles in addition to MgFe/LDH on the surface of biochar depicted successful formation of the MgFe/LDH composite with the biochar of *Ec*. Fig 1C & 1F shows that the SEM images of EDTA treated with the biochar of *Ec* and MgFe/LDH composites before and after adsorption of Pb^2+^ were shiny and porous and had aggregates with irregular structures [34].

Furthermore, to confirm the mechanism and elemental composition of MgFe/LDH and composite adsorbents, EDX spectra of pre and post adsorption of Pb^2+^ are shown in Fig 1B. The elemental content in pre adsorption (Fig 1B (a, b, c)) samples changed immediate after Pb^2+^ adsorption, as can be clearly seen in Fig 1B (d, e, f) spectra of post adsorption samples. The change in elemental content during Pb^2+^ adsorption indicates that ion exchange on the surface and in the interlayer of the synthesized adsorbent material was involved [35].

#### 3.1.2. Fourier transform infrared analysis

**Fig 2:**
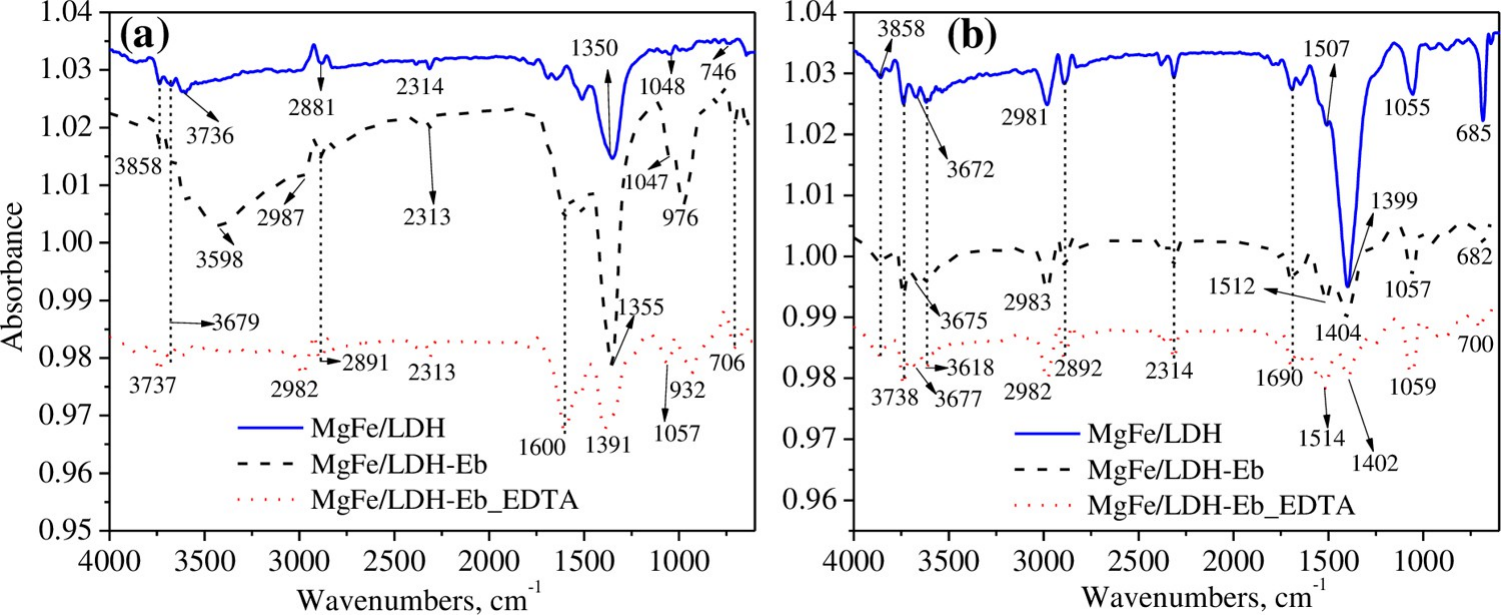
FTIR spectra of MgFe/LDH and composite adsorbents before (a) and after (b) adsorption of Pb^2+^.

The type of functional groups and the change in chemical bonding perceived on the surface of MgFe/LDH and composite adsorbents before and after the adsorption of Pb^2+^ were investigated using the FTIR technique, as shown in Fig 2. Noticeably, the spectra of all three adsorbents before adsorption showed distinct FTIR bands for the biochar, MgFe/LDH, and EDTA, as presented in Fig 2A. When MgFe/LDH was composited with the biochar of *Ec* and then functionalized with EDTA, a small peak appeared at approximately 3736 cm^-1^, and this peak moved to 3737 cm^-1^ and can be denoted as interlayer water and hydroxyl group stretching vibrations. According to the literature, LDH adsorption peaks below 3800 cm^-1^ to 3200 cm^-1^ often represent OH hydroxyl groups that are associated with the presence of H_2_O (interlayer water molecules) [33,36]. The characteristic peaks at approximately 2987 and 2881 cm^-1^ were shifted to 2982 and 2891 cm^-1^, and these were attributed to C-H groups [37]. Low intensity peaks appeared at frequencies of approximately 2314 cm^-1^ in MgFe/LDH and 2313 cm^-1^ in the MgFe/LDH-Eb and MgFe/LDH-Eb_EDTA spectra, which correspond to the C-O band related to CO_2_ [37]. Another large peak observed at a frequency of 1600 cm^-1^ in the spectra of MgFe/LDH-Eb and MgFe/LDH-Eb_EDTA is due to the O-H bending vibration from the adsorbed water molecules [36].

Other prominent peaks were observed at a frequency of 1350 cm^-1^ in the MgFe/LDH spectra, while peaks at 1355 cm^-1^ in the MgFe/LDH-Eb spectra and 1391 cm^-1^ in MgFe/LDH-Eb_EDTA corresponded to stretching vibration modes of N-O [36,38,39]. Additionally, the N-O stretching band can clearly be identified in the regions of 1500–1200 cm^-1^ and 1080–1020 cm^-1^ [39]. The very sharp peaks observed at 976 cm^-1^ in the MgFe/LDH-Eb spectra and at 932 and 706 cm^-1^ in MgFe/LDH-Eb_EDTA were attributed to M-O-H or O-M-O groups [40]. The MgFe/LDH spectra and composite adsorbents spectra after Pb^2+^ adsorption are shown in Fig 2B. Most of the absorption bands remained unchanged, or very little change in frequencies was observed. Therefore, only new absorption bands appearing after Pb^2+^ adsorption will be discussed here. A very prominent band shift was observed from 1600 cm^-1^ to 1690 cm^-1^ in all three adsorbents, and this band was ascribed to the bending vibration of water molecules [41]. New bands appearing at 1507 cm^-1^ (MgFe/LDH loaded Pb^2+^), 1512 cm^-1^ (MgFe/LDH-Eb loaded Pb^2+^) and 1514 cm^-1^ in (MgFe/LDH-Eb_EDTA loaded Pb^2+^) were ascribed to N-O stretching [39]. Another major band shift was observed at 1399 cm^-1^ in the MgFe/LDH-loaded Pb^2+^ spectra, 1404 cm^-1^ in MgFe/LDH-Eb-loaded Pb^2+^ and 1402 cm^-1^ in MgFe/LDH-Eb_EDTA-loaded Pb^2+^, which was ascribed to the N-O bending vibration of NO_3_^-^ ions. Likewise, the bands at 685, 682 and 700 cm^-1^ were ascribed to metal oxygen or hydroxyl oxygen bands (Pb-O, Pb-OH or O-Pb-O), suggesting that Pb^2+^ had adsorbed onto the tested adsorbents. Thus, these results indicated that Pb^2+^ was successfully adsorbed onto the MgFe/LDH and composite adsorbents.

### 3.2. Effects of the batch parameters on the adsorption process

**Fig 3:**
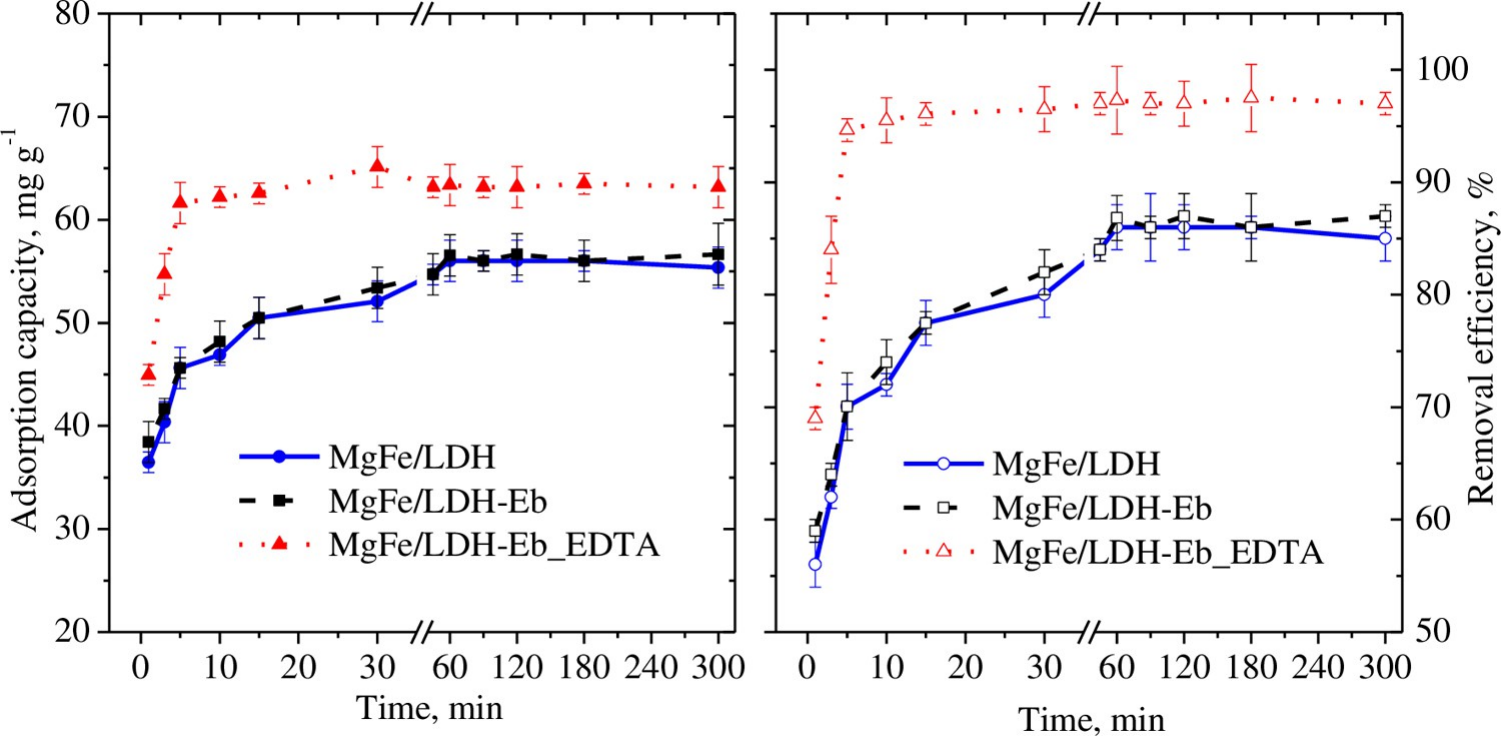
Effect of the contact time on the adsorption capacities and removal efficiencies of Pb^2+^ (20 mg L^-1^) by MgFe/LDH, MgFe/LDH-Eb, and MgFe/LDH-Eb_EDTA.

Fig 3 presents the changes in the removal efficiency and adsorption capacity of MgFe/LDH as well as the composite adsorbents at an initial concentration of 20 mg L^-1^ Pb^2+^. A very high removal of Pb^2+^ was evident upon immediate contact (more than 50% after only 1.0 min) due to the abundance of free active sites on the surface of the adsorbent. The removal rate continued to be high for up to 5 min of contact time with a greater than 90% removal efficiency for MgFe/LDH-Eb_EDTA at low initial concentrations of Pb^2+^ (20 and 30 mg L^-1^). A gradual increase in the adsorption capacity and removal efficiency was observed when the equilibrium contact time at 60 min was attained, as shown in Fig 3. No further increase in the adsorption capacity or removal efficiency was seen for any adsorbent at any initial Pb^2+^ concentration. At 60 min, the maximum adsorption capacity and removal efficiency of 63 mg g^-1^ and 97%, respectively, were seen for MgFe/LDH-Eb_EDTA, while these were nearly 55 mg g^-1^ and 86% (maximum adsorption capacity and removal efficiency) for both MgFe/LDH and MgFe/LDH-Eb, respectively, owing to the higher surface area of MgFe/LDH-Eb_EDTA compared to that of the other two adsorbents. As per multi-point BET and Barrett–Joyner–Halenda measurements, specific area of MgFe/LDH was estimated nearly 114 m^2^ g^−1^ with a pore volume and diameter of 0.37 cm^3^ g^-1^ and 3.4 nm, respectively. A high BET specific surface areas of about 127.5 and 141 m^2^ g^−1^ were measured, respectively, for MgFe/LDH-Eb and MgFe/LDH-Eb_EDTA.

**Fig 4:**
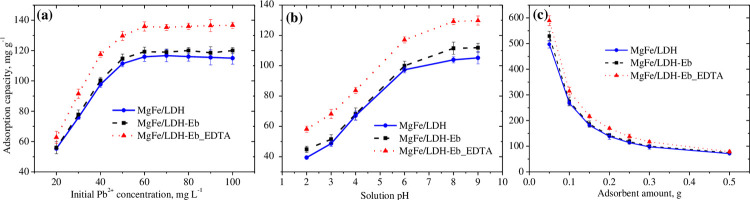
Effects of the initial Pb^2+^ concentration (a), solution pH (b), and adsorbent amount on the adsorption capacities and removal efficiencies of Pb^2+^ by MgFe/LDH, MgFe/LDH-Eb, and MgFe/LDH-Eb_EDTA.

An increase in adsorption capacity by increasing the initial Pb^2+^ concentration was observed for all three adsorbents with a maximum adsorption capacity at 60 mg L^-1^ due to the existence of a stronger driving force between the surface of the adsorbent and high Pb^2+^ in concentrated solution [42,43]. These values were recorded as 136, 119, and 116 mg g^-1^ for MgFe/LDH-Eb_EDTA, MgFe/LDH-Eb and MgFe/LDH, respectively, as shown in Fig 4A. Nearly insignificant changes (*p* = 0.01) in adsorption capacities were seen in the initial Pb^2+^ concentration range of 60–100 mg L^-1^. On the other hand, a nearly 60% decrease in the percentage of Pb^2+^ removal by all three adsorbents in the selected range of initial Pb^2+^ concentrations (20–100 mg L^−1^) is associated with the decreasing active sites of a fixed adsorbent (0.3 g) amount for high Pb^2+^ concentrations. In other words, the saturation of the active sites on the surface of adsorbents occurs due to its fixed amount (0.3 g) and thus these actives sites become unavailable for increased initial concentrations of Pb^2+^, as previously reported for different adsorption systems [44–47].

Similar trends for changes in the adsorption capacities and percentage removal were seen for all three adsorbents, wherein a significant increase in both parameters was observed as the solution pH increased from 2.0 to 6.0. In acidic conditions with solution pH 2.0 to 4.0, the low efficiency of the adsorption is resulted due to the competition between the divalent Pb^2+^ and an excess amount of H^+^ ions to attach to the negatively charged surface of the adsorbent [48,49]. Nearly 50–60% increases in the adsorption capacities and percentage removal were seen in the aforementioned range of pH values. Insignificant changes were observed in the adsorption performance as the pH increased from 6.0 to 8.0, whereas both the adsorption capacities and percentage of removal remained almost constant beyond a solution pH of 8.0. The maximum adsorption capacity at a solution pH of 8.0 was found to be 130 mg g^−1^ for MgFe/LDH-Eb_EDTA, with corresponding values of 112 and 104 mg g^−1^ for MgFe/LDH-Eb and MgFe/LDH, respectively. A reduction in the amount of H^+^ with increasing solution pH causes the divalent Pb^2+^ to attach to the surface of the adsorbent with less competition, resulting in increased adsorption capacity and percentage of removal [48–50].

A nearly similar trend of decreasing adsorption capacities and increasing removal efficiency was assessed for all three adsorbents by increasing the corresponding amount of adsorbents, as shown in Fig 4C. A maximum adsorption capacity of 589 mg g^-1^ corresponded to a percentage removal of 75% at the lowest tested amount of the composite adsorbent, MgFe/LDH-Eb_EDTA, i.e., which was 0.05 g. The corresponding maximum adsorption capacities at the same adsorbent amount (0.05 g) were recorded as 529 and 497 mg g^−1^ for MgFe/LDH-Eb and MgFe/LDH, respectively. A decreasing trend in adsorption capacities with an increased amount of the adsorbent was attributed to the availability of more free active sites for a fixed Pb^2+^ concentration (40 mg L^−1^). Although, the large surface area of the increasing amount of adsorbent is good for the efficiency of the adsorption process but the amount of the adsorbent has a direct effect on the adsorption system in terms of its cost-effectiveness, so it is always important to know the optimum value.

Considering the importance of regeneration for practical applications, the adsorption capacity of the composite adsorbent was estimated for Pb^2+^ and it was found that the amount of desorbed Pb^2+^ decreased by increasing the temperature. Moreover, a high amount of Pb^2+^ was desorbed with average desorption rate of nearly 100% at low solution pH (2.0–4.5). Finally, Table 1 provides a quick comparison for the obtained maximum adsorption capacities of the investigated adsorbents in this study with other similar adsorbents, as reported in the literature.

**Table 1:** Comparison of the estimated adsorption capacities of various adsorbents for Pb^2+^ with investigated adsorbents in this study.

Type of adsorbent	Maximum adsorption capacity, mg g^-1^ (except where mentioned)	Reference
MgFe/LDH	116	This study
MgFe/LDH-Eb	119	This study
MgFe/LDH-Eb_EDTA	136	This study
Mg_2_Al-LS-LDH	123	[51]
Kiwi branch Biochar/ZnFe-LDH	161.29	[35]
Rice husk ash/MgFe/LDH	682.2	[52]
Montmorillonite–illite type of clay	51.80	[53]
Fe_3_O_4_/GO/MgAl-LDH	173	[54]
ZnAl-LDH intercalated with EDTA	871 umol g^-1^	[55]
Mg_2_Al-LDH	66.16	[56]
ZnAl-LDH/DTPA	80 umol g^-1^	[57]
Biochar/MgFe-LDH	476.25	[58]
EDTA-LDH/Biochar	146.84	[59]
Fe_3_O_4_@SiO_2_-EDTA	114.94	[60]

### 3.3. Modeling of the experimental data using adsorption kinetics

The nonlinear expressions of the most commonly used kinetic models are expressed using Eqs 3–6. To compare the three adsorbents used, these models were applied to the adsorption data that were obtained by performing batch tests at an initial Pb^2+^ concentration of 40 mg L^-1^.


$$q_{t}=q_{e}\;(1-\exp\left(-k_{1}t\right))$$ (3)

$$q_{t}=\frac{{q_{e}}^{2}k_{2}.t}{q_{e}k_{2}.t+1}$$ (4)

$$q_{t}=\frac{1}{\beta }\ln(1+\alpha \beta t)$$ (5)

$${q_{t}=K}_{ip}t^{1/2}+C$$ (6)

Both linear and nonlinear (*q*_*t*_ vs. *t*) fitting was accomplished using the OriginPro 8.5 Software, and the estimated values (using the slope and intercept values in linear fitting) of the associated parameters in each model are listed in Table 2. In the above equations, *q*_*t*_ (mg g^−1^) is the uptake of Pb^2+^ by the adsorbent at any time *t*, while *q*_*e*_ (in Eqs 3 and 4) is measured experimentally and represents the equilibrium uptake capacity. *k*_1_ (min^−1^) and *k*_2_ (mg g^−1^ min^−1^) in Eqs (3) and (4) represent the rate constants of the pseudo-first-order (first-order) and pseudo-second-order (second-order) kinetic models, respectively. The initial adsorption rate constant and activation energy are expressed by *α* (mg g^−1^ min^−1^) and *β* (g mg^−1^), respectively, in the Elovich kinetic model (Eq 5). The rate constant and boundary-layer thickness of the intraparticle diffusion of Weber and Morris (ID-WM) kinetic model (Eq 6) are presented by *K*_*ip*_ (mg g^−1^ min^1/2^) and *C* (mg g^−1^), respectively.

**Table 2:** Estimated values of the parameters using linearized and nonlinear kinetic models for 40 mg L^-1^ Pb^2+^ onto 0.3 g of adsorbent at pH = 6 ± 0.2.

Kinetic model	Parameter	MgFe/LDH		MgFe/LDH-Eb		MgFe/LDH-Eb_EDTA	
		*Lin*	*N-lin*	*Lin*	*N-lin*	*Lin*	*N-lin*
	*q*_e exp_ (mg g^**−**1^)	97.32		99.95		117.04	
first-order	*q*_*e cal*_ (mg g^**−**1^)	10.20	92.4	10.10	94.31	9.51	112.67
	*k*_1_ (min^**−**1^)	0.010	0.52	0.006	0.54	0.011	0.47
	*R* ^2^	0.43	0.73	0.26	0.73	0.4	0.74
second-order	*q*_*e cal*_ (mg g^**−**1^)	96.15	95.96	97.09	97.69	116.28	116.84
	*k*_2_ (g mg^**−**1^ min^**−**1^)	0.0116	0.0095	0.0321	0.0101	0.0119	0.0073
	*h* (mg g^**−**1^ min^**−**1^)	107.53	87.85	303.03	96.10	161.29	99.66
	*R* ^2^	0.9999	0.95	0.9998	0.94	0.9999	0.94
Elovich	*α* (mg g^**−**1^ min^**−**1^)	3171.13	23876	6689.00	48664	2379.40	22503
	*β* (g mg^**−**1^)	7.60	0.13	7.30	0.14	9.45	0.11
	*R* ^2^	0.85	0.83	0.8	0.78	0.83	0.81
ID-WM	*K*_*ip*_ (mg g^**−**1^ min^1/2^)	2.06	2.06	1.92	1.92	2.53	2.53
	*C* (mg g^**−**1^)	71.85	71.85	74.85	74.85	86.80	86.51
	*R* ^2^	0.52	0.45	0.46	0.41	0.5	0.45

**Fig 5:**
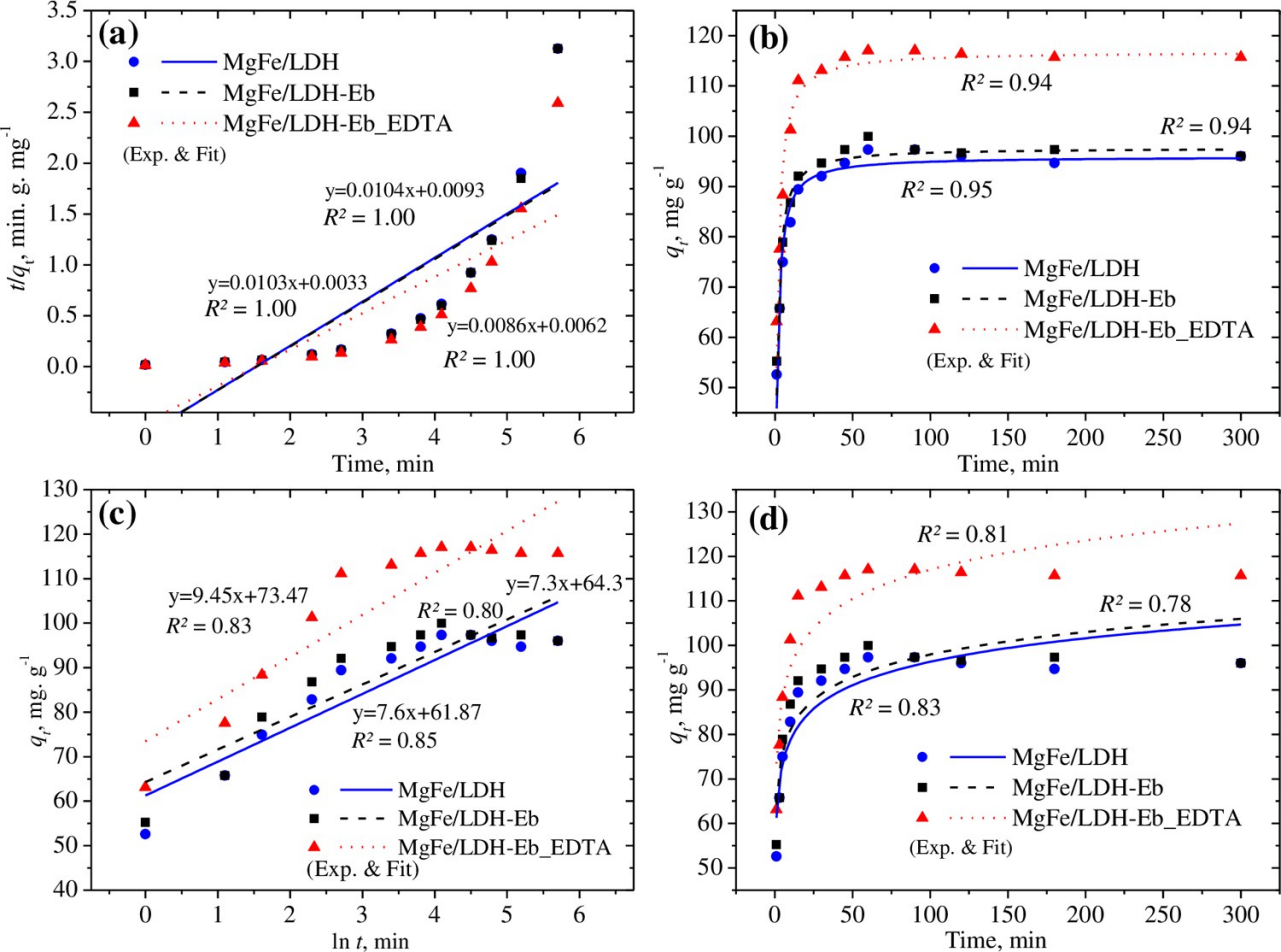
Linearized and nonlinear fitting of the second-order (a & b) and Elovich (c & d) kinetic models at 40 mg L^-1^ Pb^2+^.

Linearized and nonlinear fittings of the second-order and Elovich kinetic models to the adsorption data of Pb^2+^ (40 mg L^−1^) for all three adsorbents (0.3 g) are presented in Fig 5. A perfect linearized fitting of the second-order kinetic model (Fig 5A) is predicted based on the coefficient of determination (*R*^*2*^) value of 1.0 for all adsorbents, suggesting that chemisorption is the mechanism that controls adsorption [61–63]. The nonlinear fitting (Fig 5B) also generated high *R*^*2*^ values (0.95) for all adsorbents. Both the linearized and nonlinear fittings (Fig 5C and 5D) of the Elovich kinetic model produced nearly the same *R*^*2*^ values (close to 0.80) to fit the adsorption of Pb^2+^ on all three adsorbents.

As presented in Table 2, good *R*^*2*^ values (0.73) were recorded for the nonlinear fitting of the first-order kinetic model, and this result was further supported by a slight underestimation of the calculated maximum adsorption capacities in comparison to the experimental values (for example, 113 mg g^-1^ versus 117 mg g^-1^ for MgFe/LDH-Eb_EDTA). Approximately the same *k*_1_ (0.5 min^−1^) values for the nonlinear fitting of the first-order kinetic model were observed for all three adsorbents. A poor representation of the linearized first-order kinetic model can be predicted based on the calculated adsorption capacities and *R*^*2*^ values (Table 2) for the adsorption of Pb^2+^ onto the selected adsorbents. For the second-order kinetic model, the calculated adsorption capacities closely agreed with the experimental values for both the linearized and nonlinear fitting of the adsorption data using all three adsorbents, as presented in Table 2. The *k*_2_ and initial adsorption rate, *h*, *(k*_*2*_ *q*_*e*_^*2*^) in the nonlinear second-order kinetic model were estimated to be higher than the linearized fitting with the highest value of *h* (99.66 mg g^−1^ min^−1^) for MgFe/LDH-Eb_EDTA in the nonlinear fitting.

The ID-WM kinetic model did not yield a reasonable fit to the adsorption data of Pb^2+,^ with *R*^*2*^ values in the range of 0.4–0.5 for both the linearized and nonlinear fittings, while both associated parameters, *K*_*ip*_ and *C*, were estimated to be nearly the same (Table 2) for both linearized and nonlinear fitting for all three adsorbents, and the highest *R*^*2*^ value was observed for MgFe/LDH-Eb_EDTA (2.53 mg g^**−**1^ min^1/2^ and 87 mg g^**−**1^ for *K*_*ip*_ and *C*, respectively). The Elovich kinetic model was shown to be a better representation of the adsorption data than the first-order or ID-WM kinetic models based on the estimated *R*^*2*^ values in all three models. Both associated parameters, *α* and *β*, in the Elovich kinetic model vary considerably for the linearized and nonlinear fitting, and MgFe/LDH-Eb_EDTA had the lowest values except for *β* (9.45 g mg^−1^ as shown in Table 2) in the linearized fitting.

### 3.4. Modeling of the experimental data using adsorption isotherms

To evaluate the adsorption performance, two- and three-parameter isotherm models were applied to the experimental batch test data. A description of the commonly applicable models is provided in Table 3, and both nonlinear and linearized fittings (wherever applicable) were used.

**Table 3:** Nonlinear expressions of the two- and three-parameter isotherm models and explanation of the parameters.

Isotherm model	Mathematical expression	Parameters
Langmuir	$q_{e}=\frac{q_{m}K_{L}C_{e}}{\left(1+K_{L}C_{e}\right)}$	*q*_*m*_, maximum sorption capacity, mg g^−1^ *K*_*L*_, Langmuir constant, L mg^−1^
Freundlich	qe=KFce1n	*K*_*F*_, Freundlich constant, L g^−1^ *n*, dimensionless constant
Dubinin–Radushkevich	$q_{e}=q_{m}\exp\left(-K_{DR}\varepsilon^{2}\right)$ ε=RTln(1+1/Ce) *E* = $1/\sqrt{2K_{DR}}$	*T*, absolute temperature, Kelvin *R*, universal gas constant, 8.314 J mol^−1^·K^−1^ *E*, mean free energy of adsorption, kJ mol^−1^
Halsey	$q_{e}=\exp\left(\frac{{lnk}_{H}-lnC_{e}}{n_{H}}\right)$	*n*_*H*_ and *k*_*H*_, Halsey constants
Temkin	$q_{e}=\frac{RT}{H_{ads}}ln{(K}_{T}C_{e})$	*A*_*T*_, equilibrium binding constant, L g^−1^ *b*_*T*_, heat of adsorption, kJ mol^−1^
Harkins–Jura	$q_{e}={\left(\frac{A_{HJ}}{B_{HJ}-logC_{e}}\right)}^{\frac{1}{2}}$	*A*_*HJ*_ and *B*_*HJ*_, H–J constants
Jovanovic	$q_{e}=q_{m}[1-\exp\left(k_{j}C_{e})\right]$	*k*_*j*_, Jovanovic constant
Elovich	$\frac{q_{e}}{q_{m}}=k_{e}C_{e}\exp\left(-\frac{q_{e}}{q_{m}}\right)$	*k*_*e*_, Elovich constant
Redlich–Peterson	$q_{e}=\frac{K_{RP}C_{e}}{1+\alpha {C_{e}}^{\beta }}$	*α*, L mg^−1^ *β* (0–1), dimensionless *K*_*RP*_, R–P constant, L g^−1^
Sips	$q_{e}=\frac{q_{m}K_{S}{C_{e}}^{n}}{1+K_{S}{C_{e}}^{n}}$	*n*, degree of heterogeneity, dimensionless *K*_*S*_, energy of adsorption, L g^−1^

**Fig 6:**
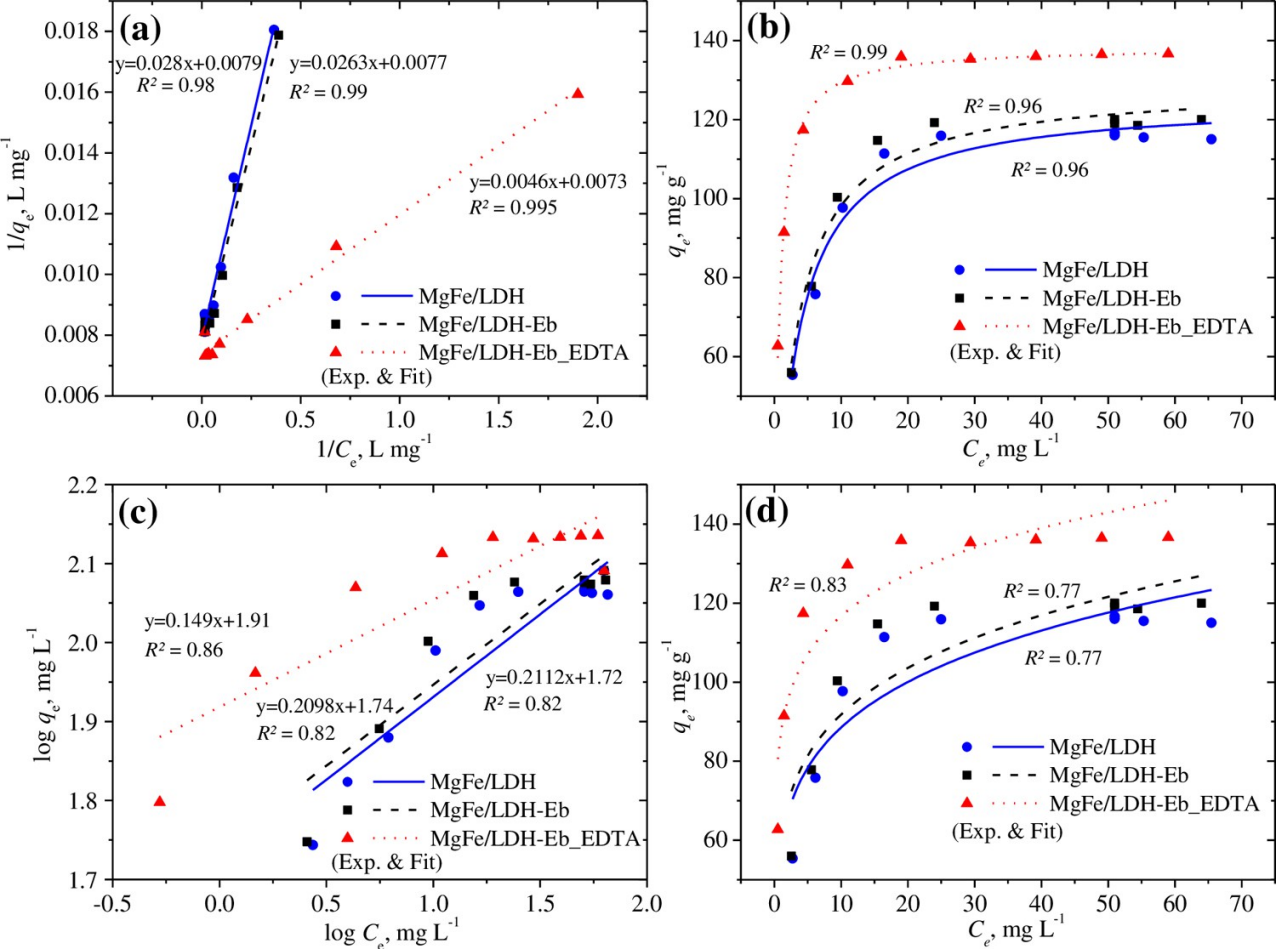
Linearized and nonlinear fitting of the Langmuir (a & b) and Freundlich (c & d) isotherm models.

Fig 6 depicts the fitting of the linearized and nonlinear fittings of the selected two-parameter isotherm models, namely, the Langmuir and Freundlich models. Slope and intercept values were used in the linearized fitting to estimate the values of related parameters at 60 mg L^−1^ Pb^2+,^ while isotherm models were fit using 20–100 mg L^−1^ of the studied metal ions with an adsorbent amount of 0.3 g. Suspensions were agitated for a contact time of 60 min by maintaining the solution pH at approximately 6.0. Slightly better fitting of the linearized model can be seen in comparison to the nonlinear fitting for both the Langmuir and Freundlich isotherm models for all three adsorbents, as predicted based on *R*^*2*^ values. The Langmuir model best presented the adsorption data with *R*^*2*^ values close to unity (1.0), which suggested monolayer adsorption had occurred, while the Freundlich model yielded *R*^*2*^ values of approximately 0.8.

As shown in Table 4, the predicted maximum adsorption capacities overestimated the experimental values for both models using all three adsorbents, although both values were closely matched for MgFe/LDH-Eb_EDTA. A better affinity between the adsorbent surface and the absorbed Pb^2+^ was predicted for MgFe/LDH-Eb_EDTA based on the *K*_*L*_ values (1.5 L mg^−1^, Table 4) in comparison to the other two adsorbents. The favorability of the Langmuir constant is further supported by the separation factor, *R*_*L*_, [(*1 + K*_*L*_*C*_*0*_)^−1^] which is in a suitable range of 0–1. The estimated values of *K*_*F*_ were highest for MgFe/LDH-Eb_EDTA (Table 4), while the calculated values of the adsorption intensity (n >1) indicated the capability of the Freundlich model [64], as presented in Table 4.

**Table 4:** Values of parameters in the linearized (Lin) and nonlinear (N-Lin) fittings of isotherm models at 60 mg L^-1^ Pb^2+^ (solution pH = 6±0.2, contact time = 60 min, and adsorbent amount = 0.3 g).

Isotherm	Parameter	MgFe/LDH		MgFe/LDH-Eb		MgFe/LDH-Eb_EDTA	
		*Lin*	*N-Lin*	*Lin*	*N-Lin*	*Lin*	*N-Lin*
	*q*_e exp_, mg g^−1^	115.89		119.23		135.89	
Langmuir	*q*_*m*,_ mg g^−1^	126.58	125.11	129.87	128.73	136.99	138.34
	*K*_*L*,_ L mg^−1^	0.28	0.30	0.29	0.32	1.59	1.45
	*R*_*L*_	0.056	0.053	0.054	0.050	0.010	0.011
	*R* ^2^	0.98	0.96	0.99	0.96	0.995	0.99
Freundlich	*q*_*m*_, mg g^−1^	139.43	123.20	144.50	123.22	161.29	148.77
	*K*_*F*_, ((mg/g)(L/mg)^1/n^)	58.72	58.96	61.21	61.43	87.63	87.37
	*1/n*	0.211	0.180	0.210	0.170	0.149	0.130
	*R* ^2^	0.82	0.77	0.82	0.77	0.86	0.83
D–R	*q*_*m*,_ mg g^−1^	111.71	113.38	115.40	117.12	130.63	132.03
	*K*_*DR*_, (mol kJ^−1^)^2^	1.0E-06	1.4E-06	1.0E-06	1.3E-06	1.0E-07	1.2E-07
	*E*, kJ mol^−1^	0.71	0.59	0.71	0.61	2.24	2.02
	*R* ^2^	0.89	0.87	0.90	0.89	0.92	0.89
Halsey	*q*_e cal_, mg g^−1^	142.90	117.77	149.16	119.09	169.48	135.38
	*n*_*H*_	-4.73	-5.67	-4.77	-5.76	-6.71	-7.99
	*K*_*H*_	0.303	0.000	0.304	0.000	0.218	0.000
	*R* ^2^	0.82	0.77	0.82	0.77	0.86	0.83
Temkin	*K*_*T*,_ L mg^−1^	13.09	13.10	14.40	14.40	281.28	281.29
	*H*_*ads*,_ kJ mol^−1^	138.12	0.05	135.57	0.05	168.51	0.07
	*R* ^2^	0.86	0.83	0.85	0.83	0.9	0.89
H–J	*A*_*HJ*_, mg g^−1^	10000	516.4	80.12	119.15	12500	840.55
	*B*_*HJ*_	3	3.9	-0.008	-0.21	2.5	4.64
	*R* ^2^	0.73	0.71	0.5	0.98	0.73	0.77
Jovanovic	*q*_*m*,_ mg g^−1^	78.03	115.58	80.12	119.15	96.53	132.75
	*k*_*j*_, L g^−1^	-0.008	-0.2	-0.008	-0.21	-0.008	-0.93
	*R* ^2^	0.51	0.97	0.5	0.98	0.43	0.91
Elovich	*q*_*m*,_ mg g^−1^	28.49		29.07		19.96	
	*k*_*e*_, L g^−1^	1.20		1.20		1.52	
	*R* ^2^	0.76		0.76		0.86	
R–P	*K*_*RP*,_ L g^−1^		25.98		28.29		214.55
	*α*, L mg^−1^		0.13		0.13		1.61
	*β*		1.12		1.12		0.99
	*R* ^2^		0.98		0.99		1
Sips	*q*_*m*,_ mg g^−1^		120.35		123.4		140.39
	*K*_*S*_, L g^−1^		0.21		0.21		1.39
	*n*_*S*_		1.28		1.33		0.88
	*R* ^2^		0.96		0.97		1

The D–R isotherm also supported the adsorption data with *R*^*2*^ values close to 0.9 and with theoretical and experimental adsorption capacities that were close (Table 4), suggesting the adsorption system was physical since the estimated *E* values were below 8 kJ mol^−1^ for all adsorbents. The Temkin isotherm model provided a better representation of the adsorption data in comparison to the Halsey, H–J and Freundlich isotherm models, as reflected by the *R*^*2*^ values (0.83–0.90); thus, it is proposed that heterogeneous Pb^2+^ adsorption occurs with uniform dispersal of binding energies on the surface of adsorbents [65]. However, the nonlinear fitting of the Temkin isotherm yielded a very low adsorption heat, while the isotherm constant was the highest for MgFe/LDH-Eb_EDTA (281 L mg^−1^, Table 4). In the case of the Jovanovic model, nonlinear fitting did improve the fitting to the adsorption data based on the *R*^*2*^ values (0.91–0.98, Table 4) in addition to generating a very close agreement for the calculated adsorption capacities and that of the experimental values for all adsorbents. A reasonable response was observed with the linearized Elovich model that explains the adsorption data based on the calculated *R*^*2*^ values; however, the theoretical adsorption capacities were greatly underestimated in comparison to the experimental values.

A very good response with both three-parameter models, Sips and R–P isotherms, was observed that explained the adsorption data, and the *R*^*2*^ values were close to unity (1.0); this reflected the homogeneous and heterogeneous adsorption of Pb^2+^ on the surface of adsorbents [66–69]. The calculated adsorption capacities were slightly overestimated in the case of the Sips isotherm model. The isotherm constant in the R–P model was much higher for MgFe/LDH-Eb_EDTA than for the other adsorbents, and it had the highest adsorption energy (1.39 L g^-1^) and lowest degree of heterogeneity (0.88, Table 4). Finally, based on the findings of isotherm models, surface adsorption seems to be less effective phenomenon as compared to the intraparticle diffusion through the internal surface of the studied adsorbents [70].

Considering the importance of the mechanistic study, results obtained from the characteristics of the loaded adsorbents are discussed to gain insights into the possible adsorption mechanisms. Heavy metal absorption and removal mechanisms using composites of various biochar and LDHs have been reported in many studies [71–73]. To comprehend the adsorption mechanism for MgFe/LDH and the composite adsorbents, samples before and after Pb^2+^ adsorption were analyzed. From all spectroscopic analysis and the results of kinetic and isothermal studies, it can be hypothesized that several mechanisms including the surface precipitation are involved in the adsorption of Pb^2+^ in this study. EDX analysis also revealed the ion exchange on the surface and in the interlayer of the synthesized adsorbent due to the change in the elemental contents of the loaded adsorbent. From the results of X-ray photoelectron spectroscopy (results not shown) slight shifting of binding energy towards the high energy side was seen after the composite adsorbent (MgFe/LDH-Eb) was loaded with Pb^2+^ and this suggests the involvement of hydroxide group in adsorption reaction due to the presence of the hydroxyl or deprotonated hydroxyl groups [74] on the surface of the loaded adsorbents. Moreover, stronger peaks in the loaded adsorbents (MgFe/LDH-Eb_EDTA) with high binding energy suggest chelation with EDTA from the LDH interlayer.

## 4. Conclusions

In the current study, the LDH of MgFe and its composites with Eb and EDTA were developed and characterized, and their potential capacity to mitigate toxic Pb^2+^ from synthetic wastewater solutions was investigated. Clumping of flowers, such as MgFe/LDH ultrafine nano glitter microspheres that were entangled with each other, was seen in the SEM images of MgFe/LDH before and after Pb^2+^ adsorption, while porous and rough surfaces of biochar particles in addition to MgFe/LDH on the surface of biochar indicated that MgFe/LDH composites with Eb were successfully formed. From the FTIR results, the appearance of new absorption bands after adsorption suggested that Pb^2+^ was successfully adsorbed onto the MgFe/LDH and composite adsorbents.

Batch tests yielded 60 min as an equilibrium contact time while a rapid removal rate of Pb^2+^ upon immediate contact with all adsorbents followed the gradual increase in adsorption capacity as well as removal efficiency. The maximum adsorption capacity and removal efficiency were 63 mg g^-1^ and 97%, respectively, for the composite adsorbent (MgFe/LDH-Eb_EDTA) due to its high surface area proposing that techniques should be employed for increasing the surface area of adsorbents and that suitable retention time be provided for optimum performance of the adsorption system. Batch testing for optimizing the initial Pb^2+^ concentration suggested 50 mg L^-1^ as an optimum value since nearly insignificant increase in adsorption capacity was seen beyond this value while removal efficiency continued to decrease. Investigation on solution pH resulted a solution pH of 8.0 with best performance using the composite adsorbent signifying the wastewater solution to be neutralized before applying the adsorption system. An opposite trend of adsorption capacities and removal efficiency by changing the amount of adsorbents makes it difficult to select the optimum amount of the adsorbent and will vary depending upon the choice of the adsorption process. A maximum adsorption capacity of 589 mg g^-1^ with highest percentage removal (75%) at the lowest tested amount (0.05 g) for the composite adsorbent (MgFe/LDH-Eb_EDTA) compared with other adsorbents signifies further the importance of selecting the adsorbent with high surface area.

A nearly perfect fitting of the second-order kinetic model based on the *R*^*2*^ value of 1.0 for all adsorbents suggested that chemisorption is the controlling mechanism of adsorption. Among the isotherm models, the Langmuir model best presented the adsorption data with *R*^*2*^ values that were close to unity (1.0); thus, the monolayer adsorption of Pb^2+^ was proposed. The D–R isotherm also supported the adsorption data with reasonable *R*^*2*^ values (approximately 0.9) and close theoretical and experimental adsorption capacities; thus, a physical adsorption system based on *E* values was proposed. The Temkin isotherm model provided a better representation of the adsorption data (with *R*^*2*^ values in the range 0.83–0.90) in comparison to that of the Halsey and H–J or even the Freundlich isotherm models, suggesting heterogeneous adsorption of Pb^2+^ occurred with uniform dispersal of binding energies on the surface of the adsorbent. Finally, an excellent representation of the adsorption data by both the Sips and R–P isotherms reflected homogeneous as well as heterogeneous adsorption of Pb^2+^ on the surface of adsorbents.
